# Medical student experiences and perceptions of palliative care in a middle eastern country

**DOI:** 10.1186/s12909-022-03448-x

**Published:** 2022-05-16

**Authors:** Halah Ibrahim, Shamsa Lootah, Karthyayani Priya Satish, Thana Harhara

**Affiliations:** 1 Khalifa University College of Medicine and Health Sciences, PO Box 127788, Abu Dhabi, United Arab Emirates; 2 Sheikh Khalifa Medical City, Abu Dhabi, United Arab Emirates; 3 Medicine Program, Kasturba Medical College, Mangalore, India

**Keywords:** Palliative care, End-of-life care, Medical students, Death and dying

## Abstract

**Introduction:**

Teaching in palliative care (PC) is an important component of medical education. Yet, studies in many countries document a fragmented and inconsistent approach to PC teaching. The goal of this study is to assess PC education, experience, and comfort levels in providing end-of-life care in recently graduated medical students.

**Methods:**

A survey was distributed to medical student applicants to residency programs at a large academic medical center in the United Arab Emirates. Descriptive statistics were used to tabulate variable frequencies.

**Results:**

Of 226 surveys, 183 were completed (80.7% response). Over half of respondents (104/183, 56.8%) did not receive any formal PC education or training in medical school. General introduction to PC (64%), pain management (68%), and non-pain symptom management (56%) were the most common topics. Only 13% (24/183) of medical students participated in PC rotations. Only 25% of participants (46/183) reported assessment of PC knowledge or skills. Gender differences were noted, with women more comfortable discussing prognosis (Pearson Chi-square value 8.67, df 3, *p* < 0.013) and assessing decision-making capacity (Pearson Chi-square value 15.02, df 3, *p* < 0.005). Few students expressed comfort with any aspect of PC. The majority of respondents (174/183, 95%) felt that it is important to receive PC education in medical school.

**Conclusions:**

Most newly graduated medical students reported limited education in PC, with minimal clinical experience. The vast majority described a lack of comfort in providing care for dying patients and their families. Educational reform is necessary to embed PC knowledge and skills into medical school curricula.

**Supplementary Information:**

The online version contains supplementary material available at 10.1186/s12909-022-03448-x.

## Introduction

Palliative care (PC) is defined as “an approach that improves the quality of life of patients and their families who are facing problems associated with life-threatening illness.” [1, 2] It is estimated that by 2060, the need for PC worldwide is expected to double, making the need for PC professionals and services a global public health concern [3]. Several studies have shown that the development and expansion of PC requires the inclusion of palliative medicine into medical school and health professional training curricula [2, 4–6]. Teaching in palliative and end-of-life care is increasingly recognized as an important component of medical education worldwide [7, 8]. Yet, studies in many countries document a fragmented and inconsistent approach to palliative medicine teaching in both undergraduate and graduate medical education, with limited clinical exposure to PC patients [9–12]. Surveys of medical students and residents consistently reveal a lack of knowledge and confidence in PC skills [9, 10]. In one study, for example, nearly half of the students in their final year of medical school felt underprepared to provide end-of-life care [13]. A systematic review of PC education in United States medical schools found significant heterogeneity in teaching methods, including lectures, elective rotations, and mandatory programs [14]. The authors noted that outcome measures improved regardless of the type or method of education [14]. Research has also shown that clinical exposure to PC and the number of times that trainees participate in caring for terminally ill patients improve overall PC knowledge, skills, and perceived competence [15].

There is limited published research from the Arab world on PC education. Existing literature has documented gaps in PC teaching in the undergraduate curriculum [11, 16]. In a recent study of medical school deans in the United Arab Emirates (UAE), barriers to PC education included limited clinical PC training facilities, lack of specialized faculty, and cultural barriers to PC education [11]. The authors noted that the medical student perspective was missing. The goal of this study is to assess PC education and clinical experience, as well as self-reported comfort levels in providing end-of-life care, in recently graduated UAE medical students. Understanding the perceptions of medical students can help inform education and policy reform in the undergraduate and postgraduate curriculum.

## Methods

### Setting

The UAE is experiencing an aging population and increase in non-communicable diseases and lifestyle-related cancers, resulting in a greater need for PC services in the country [17, 18]. A systematic assessment of PC across the Eastern Mediterranean region showed that PC was underdeveloped in the region [19]. In the UAE, limited awareness of PC by healthcare professionals and the lack of an organized educational system for PC were cited as major barriers for the advancement of PC in the country [19]. Initiatives to improve the availability of PC services are currently underway. To date, however, there are a limited number of specialized PC providers licensed in the UAE and comprehensive PC services are offered in only four hospitals [20]. Most patients receive generalist PC on inpatient hospital wards by non-specialist physicians, including medical students and residents.

### Theoretical perspective

The theoretical framework for this study is based on the essential end-of-life competencies for medical trainees, developed by Schaefer and colleagues through a survey of PC experts in the United States [21]. The domains include (1) PC principles and practice, (2) fundamentals of pain and symptom management, (3) psychosocial, spiritual, and cultural needs, (4) communication, and (5) terminal care and bereavement [21].

### Survey development

The survey instrument was developed after a comprehensive review of the literature on PC education in the undergraduate curriculum and iteratively revised by two of the authors, who have formal training in palliative medicine (TH) and medical education (TH, HI). It is based on the five end-of-life competency domains identified by Schaefer and colleagues [21]. Questions were in English and aimed to understand medical student educational experiences and comfort level providing aspects of end-of-life care within the UAE context. The instrument was pilot tested for length and comprehension on 10 current residents, with only minor textual changes made based on their comments. The final version consists of 26 questions, divided into 3 sections. Following basic demographic questions, participants were asked about PC topics covered in medical school, teaching formats used, and specific clinical experiences with palliative patients. The final section includes questions related to perceived comfort levels in providing aspects of palliative medicine on a 4 point Likert scale (1 = not at all comfortable, 2 = somewhat comfortable, 3 = comfortable, 4 = extremely comfortable).

### Data collection

Study participants included recently graduated medical school students who were applying to any of the residency training programs at a large academic medical center in the UAE. After approval by the hospital’s institutional review board, email addresses of all applicants were obtained from the institution’s education department. In January 2020, each individual received an e-mail invitation and an individual link to an online survey. E-mail reminders were sent every 2 weeks, with a total of 2 reminders. The email described the purpose of the study and explained that it was anonymous and confidential. Participation was voluntary and no incentives were offered. Consent to participate in the study was indicated by the completion and return of the survey.

### Data analysis

Data were analyzed using SPSS Statistical Software Version 26 (SPSS Inc. Chicago, USA). To assess the validity of the survey tool, convergent validity was used to determine if the survey items converged to measure a construct [22]. The percentage of total variance by each factor was calculated and a pattern matrix was used to identify the domains [22, 23]. Kiaser-Meyer-Olkin (KMO) sampling adequacy and Bartlett’s tests (to assess the strength of the relationship among the variables) were also applied. The reliability of the survey tool was tabulated using Cronbach’s alpha. Descriptive statistics were used to tabulate the frequency of the variables. Sub-group analysis was further employed to determine the correlation between the demographics and the different variables, and significance was assessed using the chi-square test. *P* < 0.05 indicated a significant difference between the variables.

## Results

### Survey validation

The validity and reliability of the PC survey instrument were determined. The consistency using the principal component analysis obtained a Kaiser-Meyer-Olkin value of 0.877, which indicated that the sampling was adequate. Bartlett’s test of sphericity was significant (0.000). Construct validity was tested through the principal component analysis, which revealed the presence of a component with an eigenvalue of 11.1, explaining 74.6% of the total variance. The overall Cronbach’s α reliability score for all 26 items for the survey instrument was 0.72, indicating good internal consistency.

A total of 226 surveys were distributed among the medical students, with 183 completed surveys returned, yielding a response rate of 80.7%. Participant demographics are listed in Table 1. More than half of respondents (104/183, 56.8%) reported that they did not receive any formal education or training in palliative or end-of-life care in medical school. For the 43% of students who did receive PC education, the topics covered and teaching formats used are listed in Table 2. Assessment was rare, with less than 25% of the participants (46/183) reporting any evaluation of their PC knowledge or skills during medical school.

**Table 1:** Participant demographics (*N* = 183)

Participant demographics	n	% N
**Gender**		
Male	60	32.8%
Female	123	67.2%
**Region of medical school**		
Gulf Cooperation Council countries	87	47.5%
Middle East	53	29.0%
Africa	1	0.5%
Asia	37	20.2%
America/Europe	5	2.7%
**Personal experience with death and dying**		
Yes	106	57.9%
No	77	42.1%

**Table 2:** Palliative care topics taught in medical school and teaching formats for PC education (*N* = 79)

Topic	% N
Pain management	67.8%
General (Introduction to palliative care)	63.9%
Non-pain symptom management (e.g. nutrition, vomiting, delirium)	50.3%
Bereavement and psychosocial support	43.7%
Advance care planning/ goal of care discussion	24%
**Teaching formats**	
Formal lectures	60.7%
Case-based learning	34.4%
Small group discussions	30.1%
Problem-based learning	32.8%
Computer-based learning	13.1%
Clerkships or clinical rotations	52.5%
Standardized patients	23.0%

Table 3 lists PC experiences during medical school. Half of the students (92/183, 50%) reported that they had never followed a PC patient for more than 2 weeks, and 58% of students (106/183) never experienced a patient’s death in an allow natural death situation. Approximately 75% of the students (137/183) admitted that they never or rarely witnessed patient-physician communication regarding a terminal prognosis.

**Table 3:** Palliative care educational experience during medical school (*N* = 183)

Items	Never	< 5 Times	5–10 Times	> 10 Times
Have you been present when a patient was pronounced dead?	25 (13.7%)	100 (54.6%)	27 (14.8%)	31 (16.9%)
Have you been present when a patient’s family was notified of a patient’s death?	41 (22.4%)	97 (53%)	24 (13.1%)	21 (11.5%)
Have you followed a terminally ill patient for two weeks or more?	92 (50.3%)	60 (32.8%)	12 (6.6%)	19 (10.4%)
Have you been present during a patient’s death in an AND or DNR (Allow Natural Death/Do Not Resuscitate) situation?	106 (57.9%)	61 (33.3%)	9 (4.9%)	7 (3.8%)
Have you been present when a patient was told of a terminal prognosis?	47 (25.7%)	90 (49.2%)	27 (14.8%)	19 (10.4%)

Results given as n (%N)

Table 4 lists students’ perceived comfort with PC. Very few students expressed comfort with any aspect of PC. Students expressed being least comfortable with informing patients and families of a terminal diagnosis (82/183, 45%) and discussing religious or spiritual aspects related to end-of-life (81/183, 44%). Only a quarter of students expressed comfort in managing end-of-life symptoms, including dyspnea (47/183, 26%) and delirium (38/183, 21%).

**Table 4:** Medical student perceived comfort levels with palliative care patient management (*N* = 183)

Clinical experience	Not Comfortable n (%N)	Somewhat Comfortable n (%N)	Comfortable n (%N)	Very Comfortable n (%N)
Informing a patient or their family of a terminal diagnosis	82 (44.8%)	70 (38.3%)	31 (16.9%)	0 (0%)
Discussing prognosis with a terminal patient or their family	66 (36.1%)	84 (45.9%)	33 (18.0%)	0 (0%)
Performing basic pain assessment	56 (30.6%)	70 (38.3%)	51 (27.9%)	6 (3.3%)
Prescribing oral opioids in palliative patients	65 (35.5%)	71 (38.8%)	44 (24.0%)	3 (1.6%)
Prescribing parenteral opioids in palliative patients	69 (37.7%)	60 (32.8%)	47 (25.7%)	6 (3.3%)
Prescribing non-opioid analgesia in palliative patients	60 (32.8%)	57 (31.1%)	56 (30.6%)	10 (5.5%)
Managing terminal delirium, anxiety and depression	81 (44.3%)	64 (35.0%)	31 (16.9%)	7 (3.8%)
Assessing and managing terminal dyspnea	65 (35.5%)	71 (38.8%)	44 (24%)	3 (1.6%)
Managing nausea, vomiting, and constipation in palliative patients	69 (37.7%)	60 (32.8%)	47 (25.7%)	6 (3.3%)
Assessing patient’s decision-making capacity	60 (32.8%)	57 (31.1%)	56 (30.6%)	10 (5.5%)
Discussing religious/ spiritual aspects related to end-of-life with patients and their families	81 (44.3%)	64 (35.0%)	31 (16.9%)	7 (3.8%)
Identifying patients’ cultural and social customs related to death and dying	91 (49.7%)	61 (33.3%)	28 (15.3%)	3 (1.6%)

In sub-group analysis, there was no correlation between comfort in end-of-life management with any prior history of educational activities or clinical exposure to palliative patients. Gender differences were noted (Table 5). Women respondents were more comfortable than their male counterparts in discussing prognosis (Pearson Chi-square value 8.67, df 3, *p* < 0.013), assessing decision-making capacity (Pearson Chi-square value 15.02, df 3, *p* < 0.005), and identifying patients’ social and cultural customs related to dying (Pearson Chi-square value 11.12, df 3, *p* < 0.026) (Table 5). The vast majority of students (174/183, 95%) felt that it is important to receive PC education in medical school. Most students (174/183, 95%) were also interested in receiving PC education during residency training.

**Table 5:** Gender differences in palliative care patient management

	Gender	Not Comfortable	Somewhat Comfortable	Comfortable	Very Comfortable	Total	*P* Value
Discussing prognosis with a terminal patient or their family	Male	30	24	6	0	183	0.013
	Female	36	60	27	0		
Assessing patient’s decision-making capacity	Male	31	18	11	0	183	0.005*
	Female	32	46	39	6		
Identifying patients’ cultural and social customs related to death and dying	Male	28	13	18	1	183	0.029
	Female	32	48	41	2		
Caring for patients at end-of-life	Male	31	13	13	3	183	0.026
	Female	40	51	30	2		
Use of parenteral opioids in palliative patients	Male	27	19	12	2	183	0.326
	Female	42	42	35	4		
Use of non-opioid analgesia in palliative patients	Male	26	17	13	4	183	0.121
	Female	34	40	43	6		

## Discussion

In this study of palliative care perceptions and experiences of newly graduated doctors in the UAE, the majority of medical students reported limited education in palliative medicine, with minimal clinical experience managing terminally ill patients. The vast majority described a lack of comfort in providing care for dying patients and their families. As there is a dearth of research on palliative or end-of-life training in the Middle East, particularly from the student perspective, our study adds to the international literature regarding the status of PC education.

Approximately 57% of students reported never receiving any PC education during medical school. Our findings are consistent with other international studies, in which medical students feel that their PC education was patchy and inadequate [9, 10, 24]. While the importance of PC training in medical school is evident, there is considerable heterogeneity in the teaching content and delivery methods, with no consensus on the best standards to teach PC [25]. Research has shown that longitudinal teaching throughout the medical school curriculum is the most effective way to improve students’ skills and comfort in providing PC [26]. Longitudinal integration can contextualize the learning process and enhance PC application in clinical practice. Incorporating PC topics into the existing curriculum, rather than as a stand-alone course, also enables medical schools to individualize the integration based on their curricular structure [27].

Survey respondents also reported limited clinical exposure to palliative patients. Over 75% of graduates reported never or rarely being present during disclosure of a terminal prognosis or during a patient’s death. Studies in the United States have reported similar findings; one study noted that 35% of students never witnessed a patient being informed of a terminal diagnosis [13]. Research has shown that medical trainees actively avoid participating in the care of dying patients [28, 29]. Studies have also found that clinical experience during clerkships has the greatest impact in improving student end-of-life care knowledge and competence [10, 30]. Increased clinical exposure is positively correlated with increased confidence [31, 32]. However, our respondents described isolated workshops that lacked strategy or cohesion and short-term rotations with limited clinical exposures to palliative patients. Only 13% of survey respondents participated in clinical PC rotations. This suggests that PC was frequently given a lower curricular priority than other aspects of medicine. As such, the vast majority of our respondents were entering residency without adequate preparation to care for terminally ill patients.

Our findings have important implications for practice and research. First, a greater focus on PC is urgently needed in the undergraduate curriculum. In the UAE, only one private medical school offers a dedicated palliative medicine course within the curriculum, and none mandate clinical rotations in PC [11]. In the Middle East and North Africa (MENA) region, only three countries, namely Jordan, Oman, and Lebanon, offer dedicated PC courses in their medical schools [19]. Egypt and Kuwait report having PC topics covered in other relevant disciplines, though limited details are available [19].

We believe that a vertical PC curriculum should be integrated into existing courses to teach patient-centered care across all years of medical school. Longitudinal education, with both didactic and clinical components, is necessary. Essential components of the curriculum include PC principles and practice, pain and symptom management, psychosocial, spiritual and cultural aspects of care, communication, and terminal care and bereavement [21]. During the pre-clinical years, basic principles of palliative and end-of-life care can be introduced as a stand-alone module, and reinforced through case-based discussions. Pain and symptom management can be taught in pharmacology and re-emphasized in courses focusing on disease-specific diagnoses and treatment. Clinical skills courses can include patient-centered communication skills around serious illness. Grief, bereavement and self-reflection on personal reactions to death and dying can be included in professional development modules. Shared decision making, ethical and legal issues in palliative care can be introduced in bioethics modules. PC training should employ mixed teaching and assessment modalities to enhance student learning and participation, including problem-based learning, group discussions, simulation and role-playing, with debriefing of clinical experiences [25, 33].

It is also vital that students put their classroom learning into practice by seeing patients with PC needs in hospital and community settings [25]. Studies suggest that PC education is more meaningful within the context of clinical training [25, 34]. Though not all medical schools can offer clinical placements in dedicated PC units, medical students participate in the care of patients with serious illness during the majority of their clinical rotations. Students can be encouraged to observe and participate in discussions with patients and families regarding terminal disease diagnosis and prognosis, goals of care, and advance care planning. Clinical teaching faculty can teach pain and symptom management and include medical students as they attend to patients’ psychosocial and spiritual needs. Role modeling of patient-centered and culturally sensitive PC communication and patient care by clinical faculty is an important component of medical student education [9, 10, 12].

The interdisciplinary nature of PC highlights the need for the curriculum to be delivered by an interdisciplinary faculty, including non-physician health professionals such as nurses, social workers, chaplains, and psychologists [25, 33]. Interprofessional teaching allows the students to appreciate each profession’s unique contribution to patient care [35]. Finally, assessment of PC knowledge and skills must be embedded into the educational program. Assessment drives learning and reinforces the importance of PC as an essential component of the curriculum [25, 36]. Multiple-choice questions, Objective Structured Clinical Examinations (OSCEs) in PC, and formal reflection writing are all methods to evaluate student PC learning [37, 38]. Several international frameworks of essential PC competencies exist for undergraduate education. While they differ in curricular components, common domains include communication with patients and families facing serious illness, principles of palliative and end-of-life care, pain and symptom management, clinical assessment of patients at the end-of-life, addressing psychological and spiritual needs, interdisciplinary teamwork, and self-care [21, 33, 34, 36]. For example, the EDUPALL project, developed by the European Association for Palliative Care (EAPC), is a blended, standardized undergraduate PC curriculum that is freely available online [34]. The curriculum covers the essential domains of PC reported in US studies [21], with an additional module covering teamwork and self-reflection, and includes learning objectives with aligned teaching modalities and assessment strategies [34]. This curriculum could be locally adapted to improve undergraduate PC education in the UAE.

Our survey did not find any correlation between clinical exposure to palliative or terminal patients and students’ perceived comfort in managing these situations. This is in contrast to several studies, which found that caring for dying patients improved PC knowledge and skills, and positively impacted comfort and confidence levels [15, 30]. It is possible that the students acted more as observers during their clinical interactions, rather than active participants in care provision, thereby minimizing any potential improvements in clinical skills. These results suggest that it is the depth and quality of clinical engagement, rather than the quantity, that influence medical student perceptions of end-of-life care.

In a recent studies in UAE medical schools and residency programs, lack of trainee awareness and interest in PC were cited as major barriers to PC education [11, 39]. Our findings, however, show high student interest in PC training, with over 95% of medical student respondents reporting that it was important to learn about PC, and 95% requesting additional PC training during residency. It is encouraging that the students recognize the value and importance of learning PC skills and are interested in further training in palliative medicine. Other research has also found that medical students want to participate in end-of-life care and find the experience to be meaningful [15, 40]. This finding should further encourage medical schools and residency training programs to integrate PC education into the curriculum.

Our findings should be viewed in light of some important limitations. First, as with any cross-sectional survey design, we cannot determine causation. We did not quantify the amount of curricular time dedicated to the specific PC topics. We also could not assess the quality of the educational or clinical training. Further, findings are based on responses from a limited number of students, and may not be representative of PC training in the UAE. The sample size potentially limited subgroup analyses. Finally, our survey was completed prior to the COVID-19 pandemic. Although clerkships were either halted or converted to virtual experiences at this time and most UAE medical students did not care directly for patients with COVID-19, it is possible that the pandemic impacted medical students’ experiences and perceptions of death and dying. Future studies on this important aspect are needed.

## Conclusions

Medical students in the UAE recognize the need for palliative medicine training, but have had limited education and clinical experience with PC and are uncomfortable providing care to dying patients and their families. As the UAE expands its medical education and healthcare infrastructure, there is a timely and important opportunity to integrate comprehensive and longitudinal PC education and training into all medical school curricula.

## Supplementary Information

**Additional file 1.**

## Data Availability

All data generated or analyzed during this study are included in this published article and its supplementary information files.
